# Baseline cultural competence in physician assistant students

**DOI:** 10.1371/journal.pone.0215910

**Published:** 2019-04-23

**Authors:** Melanie M. Domenech Rodríguez, Paula B. Phelps, H. Cathleen Tarp

**Affiliations:** 1 Department of Psychology, Utah State University, Logan, Utah, United States of America; 2 Physician Assistant Studies Department, Idaho State University, Pocatello, Idaho, United States of America; 3 Global Studies and Languages Department, Idaho State University, Pocatello, Idaho, United States of America; The University of Sydney School of Pharmacy, AUSTRALIA

## Abstract

**Purpose:**

Cultural competence is a critical component in health care services. The relationship between health disparities and prejudice and discrimination is well documented. Prejudicial attitudes and discriminatory behavior are modifiable through training yet few programs have evidence-based training. No published data has reported on baseline levels of cultural competencies in medical trainees which is necessary for tailoring programs appropriate to the audience. This manuscript fills that gap by reporting on data from three cohorts of first-year Physician Assistant (PA) students (*N* = 216). We examined students’ baseline levels with special attention to differences in cultural competence constructs across age, gender, and ethnicity.

**Methods:**

Students completed self-report measures for ethnic identity, ethno-cultural empathy, multicultural orientation, attitudes about diversity, health beliefs attitudes, colorblind racial attitudes, and burnout at the beginning of their first year. They completed the measures online (Qualtrics) during class time, prior to a lecture on cultural competence.

**Results:**

Data indicate a correlation between cultural competence constructs supporting the validity of the battery of tests as a cohesive unit to measure cultural competence. There were statistically significant differences between age, gender identity, and ethnic groups across cultural competence variables.

**Conclusions:**

Data provide baseline data that may be used to tailor educational programs. Findings suggest that our measures show promise for future educational research measuring effectiveness of cultural competence training.

## Introduction

Cultural competence is a bona fide occupational qualification for medical and mental health providers broadly [1–3]. There is overwhelming evidence that health disparities are related to prejudice and discrimination broadly [3]. And while expectations are clearly stated, little is known about how to clearly meet them in assessment [4] or training [5] activities. The purpose of this manuscript is to provide information regarding one program’s evaluation of cultural competence in their first-year students across three cohorts and examine students’ baseline levels across years with special attention to differences in cultural competence constructs across age, gender, and ethnicity. The aim of this study is to measure baseline cultural competence in first year PA students using self-measurement tools and to determine factors that may affect these baseline measures.

Dictionary definitions of culture point to beliefs, customs, ways of life, and ways of thinking or behaving shared by a people or group. In the academic literature, culture has been defined as “learned, socially shared, and variable.” [6]. The “systems of meaning” are passed on from one generation to the next and cultural groups are identifiable as cohesive social units. Culture encompasses beliefs, values, and behaviors learned by people in the context of relationships and practices shared over time. Although cultures are constantly evolving, systems of meaning remain cohesive and recognizable over time.

Cultural competence has been defined as “the ability of healthcare professionals to communicate with and effectively provide high-quality care to patients from diverse sociocultural backgrounds” [7]. Dimensions of diversity include race / ethnicity, gender, sexual orientation, religion, country of origin, among others. Most definitions of cultural competence point to three important dimensions: *self-awareness* or awareness of the self as a cultural being, *knowledge* about cultural others, and specific *skills* in working with others [8,9].

Improving cultural competence in Physician Assistant (PA) training is a professional necessity at a time when standards of competence in the health professions [3], medicine [10], and the PA profession [11] specifically require their workforce to have these skills. The principal standard of the *National Standards for Culturally and Linguistically Appropriate Services (NCLAS) in Health and Health Care* is to “provide effective, equitable, understandable, and respectful quality care and services that are responsive to diverse cultural health beliefs and practices, preferred languages, health literacy, and other communication needs.” [3]. The NCLAS Blueprint calls for training and evaluation to address this standard, but does not provide specific guidance on how to train or evaluate. The literature provides some general guidance in regards to training. A systematic review of 34 published articles found strong evidence for the impact of cultural competence training on provider’s knowledge, attitudes, skills, and patient satisfaction [5]. The link between cultural competence training and patient outcomes is weaker although emerging [12,13]. Little and incomplete information is available on cost-effectiveness of cultural competence training programs [12,13]. No reviews point to tailoring training to students’ baseline levels of competence. Finally, there is little consistency in how cultural competence is measured across studies [5,13].

Measuring cultural competence can be challenging. Measures such as Health Beliefs and Attitudes Survey [14] and the Colorblind Racial Attitudes Scale [15] have been used to examine individuals’ attitudes toward others and show strong psychometric properties. Colorblindness is the belief that race “does not matter.” [15]. Self-awareness also includes a recognition of individuals’ own cultural context. Measures examining ethnic identity [16] can provide some insights into providers’ own perception of themselves as cultural beings. Knowledge is measured by actual experiences. The Multicultural Experiences Questionnaire [17] measures multicultural experiences (including past travel, language knowledge, and friendships) and the desire to pursue more multicultural experiences. Finally, skills are possibly the most difficult to measure. Direct observations of patients and providers interacting would be ideal to examine cultural competence, however, these measures would be costly and difficult to obtain. There are no known published behavioral observation rating codes to captures cultural competence globally. In lieu of such ratings, measures that capture empathy [18] provide a good proxy for examining skills [19]. None of these measures have been used with medical trainees to inform cultural competence training development.

The present manuscript provides data across three cohorts of Physician Assistant Studies trainees. The data presented here can begin to provide context for students’ expected levels of ethnic identity, multicultural experiences, health beliefs and attitudes, and ethnocultural empathy at the outset of training and potentially inform interventions to improve cultural competence in these medical providers.

## Methods

### Procedures

The research was approved by the Utah State University Institutional Review Board (Protocol #6859). Informed consent was acquired in written form. Participants completed the battery of measures in January of 2016 (class of 2017), September of 2016 (class of 2018), and September of 2017 (class of 2019). Students completed these measures as part of an in-class exercise to increase self-awareness about cultural competence. The in-class exercise was developed in response to HRSA funding to Idaho State University to address cultural competence training and burnout prevention at the program level. Before completing the surveys, students were informed that their data might be retained for research purposes and they had the opportunity to opt in or out. This study was reviewed and approved by the Utah State University Institutional Review Board. Once students completed the surveys, the data was accessible only the first author to avoid any program faculty having access to individual’s data which could adversely affect students in the program. After the students’ scores were analyzed, individual reports were returned, and a group report was prepared and made available to students so they could review their scores relative to their peers. A sample individualized report and a group report are available through Open Science Framework at https://osf.io/rdnbj/.

### Measures

Participants completed measures for ethnic identity, ethnocultural empathy, multicultural orientation, attitudes about diversity, health beliefs attitudes, colorblind racial attitudes, and burnout. A similar battery has been utilized in a pedagogical context for multicultural training using the tripartite model [20].

#### Ethnic identity

Ethnic identity was measured with the Multiethnic Identity Measure (MEIM). The MEIM yields two subscales, exploration (5 items) and affirmation (7 items) which together comprise the full 12 item scale. Each item is answered on a scale that ranges from 1 (*strongly disagree*) to 4 (*strongly agree*). Higher scores are indicative of higher ethnic identity. The MEIM is an often-used measure of ethnic identity and has strong reliability across studies.

#### Ethnocultural empathy

The Scale of Ethnocultural Empathy [18] measures respondents empathy towards people that belong to different ethnic groups. The scale measures ethnocultural empathy along four domains of empathic feeling and expression, empathic perspective taking, acceptance of cultural differences, and empathic awareness. Each of the 30 items is rated on a scale ranging from 1 (*strongly disagree*) to 6 (*strongly agree*). The original scale developers provided evidence for the factor structure, reliability for the subscales and total scale, as well as evidence of discriminant and concurrent validity.

#### Multicultural experiences

The Multicultural Experiences Questionnaire [17] yields two indices, experience and desire, and a total score that is the sum of the two indices. The items ratings and anchors vary. Higher scores are indicative of greater experiences and desire.

#### Colorblind racial ideology

Colorblind racial ideology was measured with the Colorblind Racial Attitudes Scale [15]. The scale has 20-items and is rated from 1 (*strongly disagree*) to 6 (*strongly agree*) with higher scores indicating greater colorblindness. Items are summed and the scale ranges from 20–120. The original scale validation showed strong reliability, split-half-reliability, concurrent, discriminant, and criterion-related validity.

#### Healthcare beliefs and attitudes

Providers’ beliefs and attitudes toward patients’ opinions and cultural context were measured with the Health Beliefs Attitudes Scale (HBAS) [14]. The HBAS has four subscales (opinion, beliefs, context, and quality) and a total scale score. The wording of the items was altered to fit the PA population (e.g., “PAs should ask patients for their opinions about their illnesses”). The 15 items are rated on a Likert scale ranging from 1 (*strongly disagree*) to 5 (*strongly agree*) and the total score is calculated as a mean of items.

#### Burnout

The Abbreviated Maschlach Burnout Inventory [21,22] captured three dimensions of burnout: exhaustion, dissociation, and personal accomplishment. Items are rated on a 7-point scale from 6 (*every day*) to 0 (*never*) with higher scores on exhaustion and dissociation indicating higher burnout and higher scores on personal accomplishment indicating lower burnout.

### Data analyses

Initial examination of the variables reflected a violation of the assumption of normality. Thus we used nonparametric tests to examine correlations (Spearman Rho) and mean group differences (Mann Whitney U). To examine differences across class cohorts (graduating class of 2017, 2018, 2019) for all variables, we used Independent Samples Kruskal-Wallis tests.

## Results

Participants were first year students in a Physician Assistant Studies Department in the western United States. Students participated in assessment that were in the graduating classes of 2017, 2018, and 2019. Each year 72 students completed the assessment (*N* = 216) and a total of 204 consented to research for a participation rate of 94.44%. Students were 22 to 50 years of age (*M* = 28.71, *SD* = 5.67). Although an inclusive item asked about gender identity broadly [23], all participants identified as cisgender and predominantly female (*n* = 119, 58.3%). Students were married (*n* = 91, 44.6%), single (*n* = 61, 29.9%), in a committed relationship (*n* = 43, 21.1%), or cohabiting (*n* = 9, 4.4%). Only 3 participants reported they currently provided services to patients. The vast majority of participants identified as White American (*n* = 175, 85.8%) with the remainder identifying as Asian or Asian American (*n* = 11, 5.4%), Hispanic/Latino (*n* = 7, 3.4%), mixed ethnic (*n* = 4, 2.0%), and Black or Black American (*n* = 1, 0.5%). An additional four students selected “other” ethnicity and two did not provide ethnicity information.

Means and standard deviations across scales reveal scores below the midpoint for the exhaustion and depersonalization scales of the burnout inventory. Student scores were around the midrange for ethnic identity and colorblindness. Finally, scores were above the midrange for health beliefs, all the multicultural experiences scales, the personal acceptance subscale of the burnout inventory, and ethnocultural empathy. See Table 1 for all means and standard deviations. See Table 2 for scale ranges and midpoints.

**Table 1:** Mean and standard deviations for cultural competence constructs across groups.

		Total Sample			White Americans			Non-White Americans		
	α	*N*	*M*	*SD*	*n*	*M*	*SD*	*n*	*M*	*SD*
Ethnic Identity	.840	204	2.72	0.44	175	2.70	0.42	23	2.98	0.39
Health Beliefs	.778	203	3.97	0.42	174	3.96	0.40	23	4.08	0.51
Multicultural Experience	.726	204	27.09	5.64	175	26.71	5.56	23	30.00	5.78
Multicultural Desire	.793	204	20.83	2.93	175	20.73	2.95	23	22.13	2.03
Multicultural Experience Questionnaire Total	.762	204	47.92	7.44	175	47.45	7.36	23	52.13	6.96
Burnout: Exhaustion	.476	203	7.97	3.86	174	8.02	3.81	23	7.57	3.82
Burnout: Depersonalization	.794	203	2.74	3.45	174	2.83	3.58	23	2.26	2.44
Burnout: Personal Achievement	.631	203	13.67	3.62	174	13.66	3.60	23	14.00	3.75
Ethnocultural Empathy	.778	204	4.53	0.53	175	4.48	0.52	23	4.93	0.49
Colorblindness	.926	204	57.08	17.55	175	57.81	17.64	23	49.48	11.91

**Table 2:** Range and midpoint of variables of interest.

	Range	Midpoint	Sample Mean
Ethnic Identity	1–4	2.5	2.72
Health Beliefs	1–5	3.0	3.97
Multicultural Experience	9–42	16.5	27.09
Multicultural Desire	6–30	12	20.83
MEQ Total	15–72	28.5	47.92
Burnout: Exhaustion	0–18	9.5	7.97
Burnout: Depersonalization	0–18	9.5	2.74
Burnout: Personal Acceptance	0–18	9.5	13.67
Ethnocultural Empathy	1–6	3.5	4.53
Coloblindness	20–120	50	57.08

We tested differences between cohorts using Independent-Samples Kruskal-Wallis tests. Significant differences emerged between cohorts on multicultural experiences, exhaustion and personal accomplishment. There was a statistically significant difference in multicultural experiences, χ^2^(2) = 6.153, *p* = .046, with a mean rank multicultural experience score of 116.72 for the class of 2017, 93.80 for the class of 2018, and 96.59 for the class of 2019. There was a statistically significant difference in exhaustion, χ^2^(2) = 37.349, *p* < .001, with a mean rank exhaustion score of 118.54 for the class of 2017, 121.46 for the class of 2018, and 67.08 for the class of 2019. Finally, there was a statistically significant difference in personal accomplishment, χ^2^(2) = 52.535, *p* < .001, with a mean rank personal accomplishment score of 82.38 for the class of 2017, 79.11 for the class of 2018, and 143.24 for the class of 2019.

Next, we examined differences across White (*n* = 175) and non-White (*n* = 23) students across the variables of interest using Mann-Whitney U tests. Even though the groups were highly unequal in size and the group of ethnic minority students was quite small, significant mean rank differences were found across the two groups in (a) ethnic identity (*M*_*w*_ = 94.39, *M*_*nonw*_ = 138.41), U = 2907.50, *p* = .001, (b) multicultural experiences subscale (*M*_*w*_ = 96.08, *M*_*nonw*_ = 125.54), U = 2611.50, *p* = .020, (c) desire for multicultural experiences (*M*_*w*_ = 96.20, *M*_*nonw*_ = 124.61), U = 2590.00, *p* = .024, (d) total multicultural experiences scale (*M*_*w*_ = 95.57, *M*_*nonw*_ = 129.37), U = 2699.50, *p* = .008, (e) ethnocultural empathy (*M*_*w*_ = 93.52, *M*_*nonw*_ = 144.98), U = 3058.50, *p* < .001, and (f) colorblindness (*M*_*w*_ = 102.80, *M*_*nonw*_ = 74.37), U = 1434.50 *p* = .025. Not surprisingly, non-White students had higher rank means for ethnic identity, multicultural experiences, ethnocultural empathy, and lower mean rank colorblindness than White students. There were no differences across groups in health beliefs or the burnout scales. See Table 1 for sample means and standard deviations.

An examination of mean differences across gender using the Mann Whitney U test revealed three statistically significant differences between men and women in the sample. Men had lower mean rank ethnocultural empathy scores than women (*M*_*w*_ = 110.19, *M*_*m*_ = 91.73), U = 4142.00, *p* = .028. Women had lower mean rank colorblind scores than men (*M*_*w*_ = 93.93, *M*_*m*_ = 114.50), U = 6077.50, *p* = .028. Women had lower mean rank depersonalization scores than men (*M*_*w*_ = 92.19; *M*_*m*_ = 115.61), U = 6172.00, *p* = .003.

Since age is a continuous variable, relationships between age and the cultural competence constructs were examined via Spearman Rho correlation (see Table 3). Two statistically significant relationships were evident between age and ethnic identity, and age and multicultural experiences. The negative correlation between age and ethnic identity (*R* = -.203, *p* = .004) shows that younger students tend to report a stronger sense of identity compared to older students. Conversely, older students tend to report more multicultural experiences than their younger counterparts (*R* = .222 *p* = .002).

**Table 3:** Correlations between cultural competence constructs.

	1	2	3	4	5	6	7	8	9	10
*n*	199	204	203	203	204	204	203	203	203	204
1 Age in years	1									
2 Ethnic identity	-.203**	1								
3 Health beliefs	-.044	.161*	1							
4 Multicultural experience	.222**	.138*	.125	1						
5 Multicultural desire	.095	.133	.304**	.380**	1					
6 MEQ Total	.211**	.153*	.214**	.923**	.683**	1				
7 Exhaustion	-.033	.128	.016	.044	-.030	.031	1			
8 Depersonalization	.083	.026	-.191**	-.030	-.110	-.062	.486**	1		
9 Personal Achievement	-.07	.13	.091	-.036	.007	-.037	.054	.076	1	
10 Ethnocultural empathy	.136	.141*	.361**	.378**	.443**	.459**	-.051	-.092	.124	1
11 Colorblindness	-.074	.169*	-.199**	-.150*	-.282**	-.221**	.046	-.049	-.078	-.550**

Note:

* = *p* < .05

** = *p* < .01

We also examined the cultural competence constructs to verify that they were related in the ways in which we expected (see Table 3). Significant negative relationships were observed between colorblindness and health beliefs, multicultural experience, desire, and total scores, and ethnocultural empathy such that students with lower colorblind scores had (a) more positive attitudes toward culturally diverse patients in the medical setting, (b) a greater desire to have multicultural experiences, and (c) higher empathy for persons of diverse ethnic groups. The colorblindness construct was not related to any of the burnout scales. See Table 3 for strength of correlations and statistical significance.

Ethnocultural empathy showed strong relationships with health beliefs, multicultural experiences, multicultural desire, and the total MEQ scale such that students with higher ethnocultural empathy scores had (a) more positive attitudes toward culturally diverse patients in the medical setting, (b) a greater number of multicultural experiences, and (c) a greater desire to have multicultural experiences. There were no significant relationships between ethnocultural empathy and the burnout. See Table 3 for strength of correlations and statistical significance.

Health beliefs and attitudes that are patient-centered and consider the patient's culture were also strongly correlated with multicultural desire and total score, in addition to the already reported relationships with ethnocultural empathy and colorblindness. Specifically, students with more culturally centered health beliefs and attitudes reported significantly higher desire for multicultural experiences as well as more frequent multicultural experiences than students with lower health beliefs and attitudes. There was also a strong negative correlation between culturally centered health beliefs and attitudes and depersonalization. See Table 3 for strength of correlations and statistical significance.

## Discussion

PA students show relatively low levels of burnout, average colorblindness [15], high levels of ethnocultural empathy, personal acceptance, and health beliefs. These scores make sense in the context of students at the beginning of their graduate program and, although relatively good, there is evidence of room for improvement. Furthermore, important differences emerged across groups by age, gender identity, and ethnic minority status that are important for programs to consider. Structural diversity, that is accepting students who are from different age, gender identity, racial/ethnic, and other backgrounds, may play an important role in bringing diversity of thought and experience into the classroom. This is consistent with established findings on the relationship between structural diversity and classroom learning [24]. Representation of ethnic minorities in PA studies is already low [25] and was even lower than expected in our sample. One simple way to intervene to increase cultural competence may be to work to change the composition of the student body along gender and ethnic lines. That can be an incredibly challenging task, especially in rural locations that have little ethnic diversity in surrounding communities.

Existing health disparities and professional mandates to work proactively to reduce them, require immediate attention to increasing cultural competence in medical education and cannot be paused while student bodies become more diverse. The health professions follow evidence-based approaches to services provision across specialty areas. It is surprising that in the literature documenting cultural competence development little attention has been paid to obtaining data about the population being trained that could support the development of appropriate training (intervention) activities. Trainings are simply developed and delivered without a strong foundation of knowledge of the group being trained. We report on a battery of measures related to cultural competence that show good reliability when used with PA students. Students across three cohorts were able to complete the battery of surveys in about 20 minutes and provided important data for program purposes. It may be very useful to collect data at multiple points in time over training to gain an understanding of how these variables move over time. For example, there is a documented relationship between empathy and burnout termed compassion fatigue [26]. It makes sense that these relationships are not evident in our data, a mere month into training. Overall, cultural competence constructs can be measured with acceptable reliability in a sample of PA students.

For the most part, scores across cohorts were stable. However, we found some differences by cohort multicultural experiences and two burnout scales, exhaustion and personal accomplishment. These may be a function of differences in the timing of data collection. For the 2017 cohort, data collection occurred early in the spring of their first year instead of the fall, as was the case for the 2018 and 2019 cohorts. When they took the self-assessment, some PA students in the 2017 cohort had already participated in community and short-term medical service trips as part of their training. On the burnout scales, it may be that one semester into PA studies, students are already beginning to show some signs of distress. It may be useful in training to assess burnout at various times during training to assess the progression and, perhaps more importantly, give students an indication of their scores so they may act to reduce distress. This is consistent with research that support self-monitoring in the attainment of goals ([27]

More diverse programs may find different baseline levels of cultural competence in their student cohorts. Samples with larger numbers of ethnic minority students would provide more robust and stable findings from which to build. Our data are also cross-sectional and self-reported. Advancing our knowledge of cultural competence should include experimental and/or longitudinal research. The limited ethnic diversity in our sample results in limited generalizability of our findings and signals the need for more data on PA students of color.

The significant correlations between cultural competence constructs suggests that we were measuring variables that are conceptually related. The different scales had strong relationships between them suggesting that the constructs may be dimensions of a larger concept (i.e., cultural competence), yet the relationships were not so strong so as to suggest we were measuring a single construct. Each scale provided unique information. Future research should focus on developing cutoff scores for these scales so that scores may be used to determine the level of training needed for specific cohorts, and, potentially, to also identify needed targeted training for students that are significantly below their peers.

### Implications to current educational practice and future research

Since our data are correlational, causation should not be implied. However, from a conceptual standpoint, some constructs are more “movable” than others. For example, programs can easily provide opportunities for engagement in multicultural experiences. Faculty and staff modeling of multicultural engagement may be powerful in moving students desire for multicultural experiences. Another relatively accessible point of intervention is with colorblindness. Consistent with existing recommendations from the Tool for Assessing Cultural Competency Training [28], instructors can target colorblindness in the curriculum by including content that makes culture visible (e.g., reporting health disparities). Furthermore, programs can use these scales with their students in an effort to increase students’ self-awareness in regards to the various constructs and also their movement over time [20]. Connecting the interventions to specific constructs being measured would allow for a clear connection between interventions and the accompanying shifts in specific cultural competence constructs providing medical educators with a process befitting an evidence-based profession.

## Conclusions

Culture and language are critical to how health care services are delivered and received, as they define the limits and effectiveness of the working relationship between the health care provider and the patient. If health professions programs wish to produce more providers who choose to work with underserved populations, it is imperative that they prepare students who are sensitive to the needs and preferences of culturally diverse patients. Overall, this manuscript contributes important knowledge regarding baseline levels of various cultural competence constructs in three cohorts of PA students. As the field tries to address the need for cultural competence training and, with it, the assessment of training efforts, these data suggest that our measures show promise for future use.
